# Investigating the Interaction Between Form and Motion Processing: A Review of Basic Research and Clinical Evidence

**DOI:** 10.3389/fpsyg.2020.566848

**Published:** 2020-10-30

**Authors:** Rita Donato, Andrea Pavan, Gianluca Campana

**Affiliations:** ^1^Department of General Psychology, University of Padua, Padua, Italy; ^2^Human Inspired Technology Research Centre, University of Padua, Padua, Italy; ^3^Department of Psychology, University of Bologna, Bologna, Italy

**Keywords:** glass patterns, motion-form integration, ventral and dorsal stream, neural modulation, conscious perception

## Abstract

A widely held view of the visual system supported the perspective that the primate brain is organized in two main specialized streams, called the ventral and dorsal streams. The ventral stream is known to be involved in object recognition (e.g., form and orientation). In contrast, the dorsal stream is thought to be more involved in spatial recognition (e.g., the spatial relationship between objects and motion direction). Recent evidence suggests that these two streams are not segregated but interact with each other. A class of visual stimuli known as Glass patterns has been developed to shed light on this process. Glass patterns are visual stimuli made of pairs of dots, called dipoles, that give the percept of a specific form or apparent motion, depending on the spatial and temporal arrangement of the dipoles. In this review, we show an update of the neurophysiological, brain imaging, psychophysical, clinical, and brain stimulation studies which have assessed form and motion integration mechanisms, and the level at which this occurs in the human and non-human primate brain. We also discuss several studies based on non-invasive brain stimulation techniques that used different types of visual stimuli to assess the cortico-cortical interactions in the visual cortex for the processing of form and motion information. Additionally, we discuss the timing of specific visual processing in the ventral and dorsal streams. Finally, we report some parallels between healthy participants and neurologically impaired patients in the conscious processing of form and motion.

## Introduction

**FIGURE 1:**
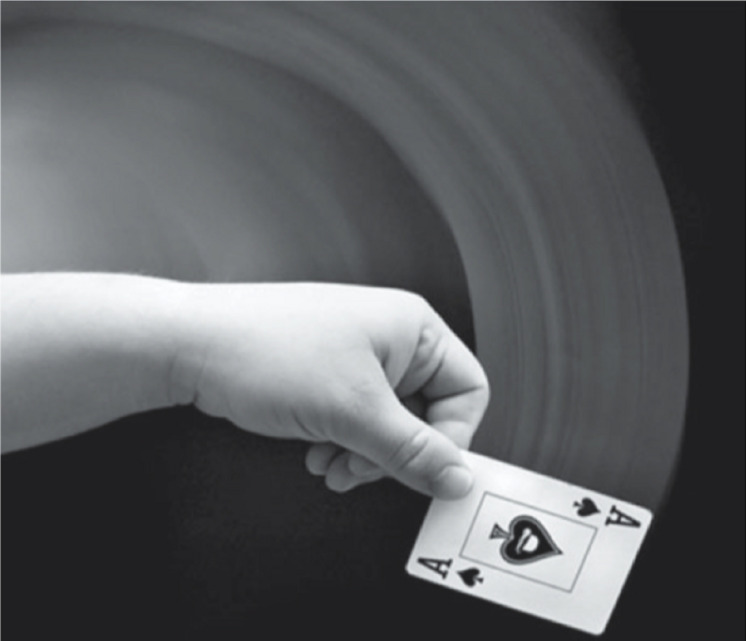
Example of motion streak reported by Apthorp et al. (2013). The blurred line that precedes the object (the playing card) gives an index of motion direction. Photo credit: Tod Klassy.

It is generally claimed that motion perception in visual stimuli involves sensors selective to direction, while form perception involves neurons selective to orientation and size (Mishkin et al., 1983; Ungerleider and Haxby, 1994; Braddick et al., 2000). The visual system is organized in two anatomically distinct pathways: the dorsal stream and the ventral stream. The dorsal stream connects the striate cortex and the parietal area. It is known as the “where” stream because it is involved in motion, spatial processing, and goal-directed actions. On the other hand, the ventral pathway connects the striate cortex and the inferior temporal area, and it is known as the “what” stream because it processes form information (Shen et al., 1999; Figure 1). Ungerleider and Mishkin (1982) were the first authors to propose the idea that the cortico-cortical projections that originate from the striate cortex are organized into two functionally distinct and anatomically separate streams.

However, there is evidence that the dorsal and ventral streams are not independent and separate as initially thought but that they constantly interact to provide a uniform and stable representation of the visual scene.

**FIGURE 2:**
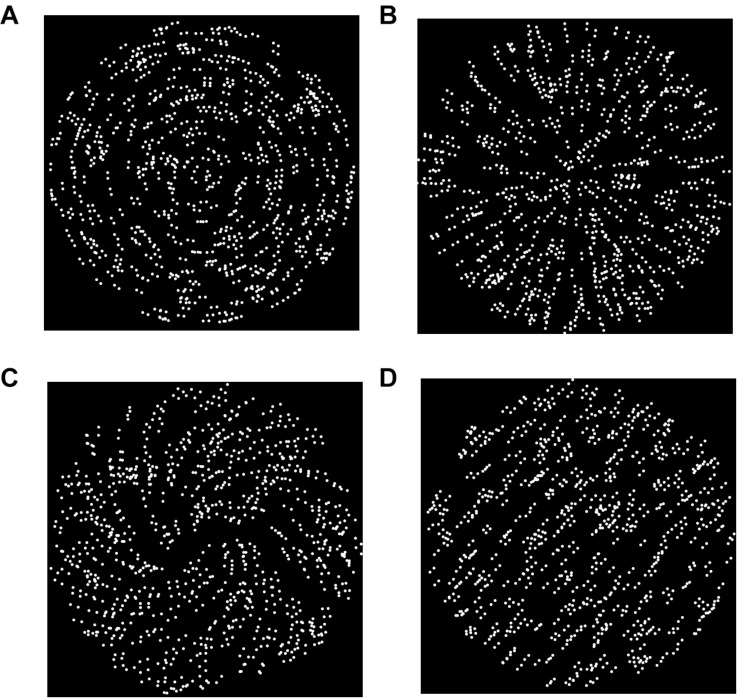
Common types of static GPs used in studies that investigate the neural basis of form and motion interaction. **(A)** Circular GP; **(B)** radial GP; **(C)** spiral GP; **(D)** translational/parallel GP.

An example of form and motion interaction comes from the studies on “*motion streaks*” (Geisler, 1999). Motion streaks are blurred lines created by fast motion that follow the object’s trajectory. This phenomenon is due to the persistence of the retinal image, and it usually aids the processing of directional motion (Figure 2; Burr and Ross, 2002).

A peculiar feature of motion streaks is that they are speed dependent (Apthorp et al., 2009, 2013; Alais et al., 2011). In a set of psychophysical experiments conducted by Geisler (1999), the author found that participants reported higher detection thresholds when the motion of a Gaussian dot (target) was masked by parallel dynamic random lines (noise) compared to the condition in which the noise was perpendicular to the direction of the Gaussian dot. However, this result was obtained only when the speed threshold was approximately one dot width per 100 ms. This phenomenon occurs because cells in the early visual cortex have a temporal integration window of ∼100 milliseconds (Burr, 1980; Snowden and Braddick, 1989; Apthorp et al., 2009; Alais et al., 2011); consequently, rapid moving objects produce motion smear.

According to Geisler’s model (Geisler, 1999), the visual system uses the orientation information created by the partial smearing of an object to infer its trajectory. Therefore, orientation-selective neurons contribute to improving the perception of motion direction. Geisler’s model might be considered an example of interaction of the dorsal and ventral streams that strengthens the encoding of “motionness” of visual stimuli (Kourtzi et al., 2008; Or et al., 2010; McCarthy et al., 2012; Apthorp et al., 2013; Mather et al., 2013; Blair et al., 2014; Tang et al., 2015; Pavan et al., 2017b). This means that the orientation information that is thought to be processed by the ventral stream facilitates the perception of motion direction that is known to be processed by the dorsal stream. Geisler’s model goes beyond the classical view of the visual system that considers motion and form as two segregated and distinct processes (Ungerleider and Mishkin, 1982). There is neurophysiological evidence in cats and monkeys that motion streaks are processed by both motion and form selective areas in the visual cortex (Geisler et al., 2001). This was also confirmed by a neuroimaging study of Apthorp et al. (2013). The authors used fMRI to measure brain activity while participants observed either fast random-dot stimuli drifting at 13.02 deg/s (i.e., inducing motion streaks), slow random-dot stimuli (1.63 deg/s) moving in different directions, or static-oriented stimuli. The authors found patterns of brain activity in early visual cortical areas distinguishing between different static orientations. A multivariate pattern classifier trained on the brain activity evoked by the static oriented stimuli could then distinguish the direction of fast (i.e., *motion streaks*) but not slow motion. The authors found that the early visual cortex was activated for fast motion, while human area MT (hMT +) responded similarly to fast and slow motion. This indicates that fast directional motion that elicited “*motion streaks*” was not only processed by the motion complex (hMT +), as reported by early cell recording studies, but also by the primary visual cortex sensitive to the oriented cues created by high-speed motion.

The role of the area MT in visual motion processing was firstly studied by Allman and Kaas (1971) in owl monkeys through a set of experiments that explored the visuotopic organization of the middle temporal gyrus. Subsequently, Zeki (1974) identified in macaque monkeys an equivalent visual motion area in the superior temporal sulcus (STS). Recent researches are now focused on studying not only motion processing but also how form and orientation signals can influence the perception of motion; motion streak studies are an example.

Ross et al. (2000) also provided experimental evidence in support of the motion streak hypothesis. The authors used a class of visual stimuli called Glass patterns (GPs; Glass, 1969) that are formed by pairs of dots known as *dipoles* to demonstrate that form cues give the percept of coherence to an incoherent motion. Different and complex shapes can be obtained from GPs depending on the geometric transforms applied to the local orientation of dipoles. The aim of the experiment of Ross et al. (2000) was to assess the limits of the perceived spin in dynamic GPs. In particular, they used dynamic circular GPs made by ten independent frames and asked participants to judge from 0 to 9 whether the circular motion they perceived was clear and vivid or not. Although participants saw circular motion in GPs, the perceived motion direction was ambiguous (i.e., whether the motion was clockwise or counter-clockwise). Comparing participants’ performance in discriminating the spin of circular GPs and the spin of real rotational motion, created by simple directional dots, participants showed similar performance for both types of visual stimuli. This indicates that the visual system does not distinguish between apparent and real circular motion. In conclusion, Ross et al. (2000) demonstrated that the orientation (form) signal contained in GPs influences the perception of motion direction because, in dynamic GPs, the perception of motion is not generated by directional motion signal, but it is created from internal form cues. An additional study with GPs that supports the motion streak hypothesis was performed by Burr and Ross (2002). Their study aimed to demonstrate how motion streaks facilitate the perception of motion direction when streaks are aligned with motion trajectory or alter motion direction when they are oriented incongruently with respect to the motion trajectory. The authors used GPs as visual stimuli and the bandpass-limited noise-masking technique. The experiment consisted in showing participants discontinuous motion in correspondence to the presentation of either oriented noise or GPs. The results showed that when the orientation of the narrow-band noise corresponded to motion direction, participants had a worse performance in motion direction detection. This can be explained considering that the noise masked the motion streak created by directional motion, desensitizing orientation processing. Moreover, GPs discrimination showed that dipole orientation could either influence the apparent motion direction when both are congruent, as demonstrated by Ross et al. (2000), or impair participants’ performance when dipole orientation supplies an apparent false direction, for example by randomly varying dipole orientation in a range of orientations between −20 to + 20 deg from horizontal (Burr and Ross, 2002). Considering the aforementioned studies, it becomes clear that GPs are an ideal class of visual stimuli to study form and motion processing. In the following paragraphs, we report in detail the spatial and temporal features of GPs that allow us to investigate the mechanisms of form and motion integration. In particular, we focus on the mechanism underlying the perception of GPs as directional moving patterns with no apparent directional motion, clinical-based research with GPs, non-invasive brain stimulation assessing form and motion processing, and the feedforward and feedback connections that contribute to the encoding and integration of form and motion signals.

## Glass Pattern Overview

**FIGURE 3:**
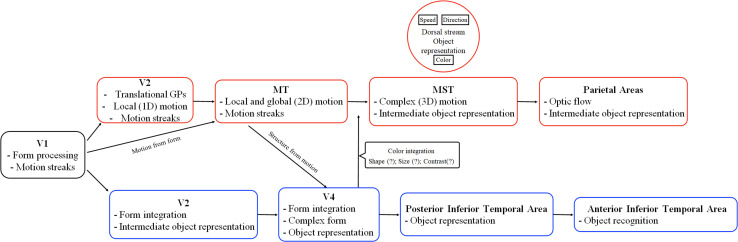
Schematic representation of the ventral and dorsal stream functions according to state of the art starting from V2. The red boxes report some areas and functions of the dorsal stream, where-as the blue boxes report areas and functions of the ventral stream. Adapted from Perry and Fallah (2014).

Glass patterns (GPs; Glass, 1969) are composed of an ensemble of dot pairs (*dipoles*) randomly displayed in a specific window. Different types of geometrical transforms can be applied to the dipoles to induce the perception of a variety of shapes, such as a translational/parallel (i.e., oriented) GPs, radial, spiral, or concentric GPs (Figure 3).

The global orientation of a GP depends on the local orientation of the dipoles that follows specific geometric rules (Chen, 2009). GPs are an important tool to study how and where the visual processing of global form of different complexity takes place. GPs can be either static or dynamic. Static GPs are characterized by only one video frame, while dynamic GPs are characterized by a rapid sequence of frames with no dipole-to-dipole correlation between consecutive video frames (Nankoo et al., 2012; Pavan et al., 2017a). Despite the lack of dipole directional motion, dynamic GPs activate motion-sensitive areas and induce the perception of non-directional apparent motion (Ross et al., 2000; Krekelberg, 2005b; Ostwald et al., 2008; Or et al., 2010; Pavan et al., 2017a,b).

Pavan et al. (2017a) argued that the perception of dipole orientation in dynamic GPs is influenced by Geisler’s (1999) spatial motion sensor; that is, the information coming from motion sensors integrated with the signals from orientation-selective units. Apparent motion direction in dynamic GPs is perceived according to the orientation of dipoles. For this reason, dynamic GPs have been used to investigate the neural basis of form and motion processing and, in particular, to assess the interaction between the ventral and dorsal streams.

The perception of both static and dynamic GPs involves two main processing stages: firstly, the local processing that allows the detection of the orientation of the single dipoles, and secondly, the global processing that enables the extraction of the overall shape from the pooling of local orientation cues (Prazdny, 1984; Wilson and Wilkinson, 1998; Pei et al., 2005; Chen, 2009; Chung and Khuu, 2014).

## Probing Form and Motion Cues: Effects of Stimulus Configuration

Several studies explored the differences in the detection thresholds of various types of GPs, suggesting that the human visual system differently discriminate GPs based on their global form (Wilson and Wilkinson, 1998; Morrone et al., 1999; Dakin and Bex, 2001; Seu and Ferrera, 2001; Chen, 2009; Nankoo et al., 2012; Pavan et al., 2019).

Wilson and Wilkinson (1998) found that static GPs with a circular orientation are the easiest to perceive, while parallel GPs are the most difficult. This result was confirmed by Lee and Lu (2010), who found lower detection thresholds for concentric and radial GPs rather than translational GPs. The evidence clearly shows that human participants perceive more easily complex GPs (e.g., circular and radial) than simpler oriented GPs (i.e., parallel/translational). Seu and Ferrera (2001) investigated the detection thresholds of circular, radial, and spiral GPs. They showed that circular and radial GPs are easier to perceive than spiral GPs, with spiral GPs having intermediate coherence thresholds between radial and circular patterns.

In a recent study, Rampone and Makin (2020) found a similar pattern of results. The authors measured the differences in brain responses for static translational, circular, and radial GPs using EEG and event-related potentials (ERPs). In particular, they measured the sustained posterior negativity (SPN) that is an ERP, indicating the perceptual goodness of various geometric regularities. Perceptual goodness is a concept introduced by Gestalt psychologists to refer to what they called “*gute Gestalt*,” i.e., “good shape.” Therefore, perceptual goodness indicates a visual perceptual experience characterized by regularities, simplicity, and order (Pashler, 2002). Perceptual goodness is also influenced by the number of parts of the object: as the number of parts of the object increases, the number of sides increases as well and consequently the overall complexity of the object. Wide SPN amplitudes point to high perceptual goodness regularities of the visual stimulus (Makin et al., 2016). Rampone and Makin (2020) studied the perceptual goodness in various types of GPs, and they found that observers had similar SPN for circular and radial GPs but not for translational GPs. Finally, observers reported weak responses to translational GPs, suggesting that translational GPs were more difficult to identify.

Chen (2009) investigated the detection thresholds of different static GPs using masking, which is useful to explore the coding efficiency of different GP types. In particular, the author tested whether the global perception of a target GP (either a radial GP or a concentric GP) was disrupted by the presentation of another GP (the masker). Seven types of masking GPs were used: noise (0% of coherence), radial, vertical, plaid, concentric, and spiral. The author investigated the detection thresholds of the GPs and observed the differences in the target threshold according to the masker used. The results showed that for concentric targets, concentric and spiral maskers had the best masking effect, whereas for radial targets, a low-curvature spiral mask produced the higher masking effect. Moreover, measuring the detection thresholds of the different GPs, circular GPs were easier to perceive than radial GPs.

A different pattern of results is obtained for the detection of real/directional motion with distinct trajectories created by random dot kinematograms (RDKs). In this regard, there are two studies of Blake and Aiba (1998) and Morrone et al. (1995) who found that there are no differences in the detection thresholds of translational, concentric, or radial trajectories evoked by RDKs. The only difference in the detection thresholds of directional RDKs concerns spiral trajectories, for which detection thresholds are higher than for the other moving patterns (i.e., circular, radial, and translational motion) (Morrone et al., 1999).

Nankoo et al. (2012) investigated whether, using various stimulus configurations (concentric, horizontal, radial, spiral, and vertical patterns), the detection thresholds of dynamic GPs were more similar to the detection thresholds of RDKs or to those of static GPs. In general, the detection threshold of dynamic GPs is lower than those of static GPs (Burr and Ross, 2006; Or et al., 2010). Nankoo et al. (2012) aimed to assess whether detection thresholds of dynamic GPs are lower than those of static GPs because they are processed by motion-sensitive units or because their processing relies on mechanisms that drive static GP perception. The interesting result was that dynamic GPs were perceived more similarly to static GPs than RDKs because the detection thresholds of dynamic GPs had a similar trend to those of static GPs rather than to those of RDKs. This outcome suggests that the stimulus information in dynamic GPs is integrated throughout the various independent frames; therefore, dynamic GPs seem to rely on temporal summation processes. Moreover, the authors found that participants were particularly sensitive to concentric and radial GPs and discriminated better vertical GPs than horizontal GPs (either dynamic or static), a phenomenon called “horizontal effect.” This effect was also found and explained by another research conducted by Hansen and Essock (2004) who demonstrated that in natural images, it is more common to see a vertical organization than a horizontal pattern. Nankoo et al. (2012) found a different trend for RDKs; in fact, participants did not show any significant difference in the detection threshold for concentric, horizontal, radial, and vertical RDKs, but they found a higher detection threshold for spiral RDKs, although spiral GPs were perceived as relatively good. In summary, despite Nankoo et al. (2012) found a similar detection threshold between different static and dynamic GPs, dynamic GPs showed generally lower detection thresholds than static GPs. An explanation of this result is that in dynamic GPs, form and motion information is integrated. The difference between dynamic GP and RDK detection threshold suggests that dynamic GPs are primarily perceived and processed for their global form and the involvement of the motion system steps in afterward.

Additionally, Nankoo et al. (2015) investigated whether the lower detection thresholds found in dynamic GPs depend on a temporal summation process of form signals. Considering that dynamic GPs are made up of different and independent static GPs presented on consecutive video frames, they possess multiple global form information. To test this hypothesis, the authors measured participants’ detection thresholds for translational static GPs and dynamic translational GPs by manipulating the number of unique frames and the consequent temporal frequency of the dynamic patterns. The authors expected lower detection thresholds for dynamic GPs with a higher number of unique frames, providing that form signals are summed over frames. Moreover, they used only translational GPs because, in their previous study (Nankoo et al., 2012), they found that the difference in participants’ detection threshold between static and dynamic GPs was greater for translational GPs compared to other types of GPs. Therefore, the usage of translational GPs should provide high statistical sensitivity as the numbers of unique frames and the temporal frequency vary. The task consisted of a two-alternative forced-choice task where participants had to indicate in which time interval the coherent translational GP was presented. The lowest detection threshold was obtained in the dynamic condition with the highest number of unique frames (i.e., 12). Although the results confirmed the hypothesis of temporal summation across unique frames of GPs, the authors did not exclude that motion processing mechanisms might contribute to the coding of dynamic translational GPs.

The integration between form and motion processing seems to play an essential role in the creation of a coherent image of the dynamic environment that surrounds us (Kourtzi et al., 2008). In order to explore the existence of form and motion interaction, Pavan et al. (2017b) conducted a study with dynamic-oriented GPs. In the experiment, the authors varied the angle between dipole orientation and dipoles’ motion direction (“*conflict angle*”). In their first experiment, the speed of the moving dipoles was likely to limit the presence of motion streaks, whereas in the second experiment the speed was adjusted to promote the presence of orientation signals from motion (i.e., motion streaks). In separate experimental sessions, participants had to report either the perceived motion trajectory or the global orientation of the dynamic GPs. The results showed that apparent GP motion direction is attracted toward dipole orientation, and GP orientation is repulsed from GP motion. Additionally, the authors found stronger repulsion effects when judging the GP orientation; however, stronger motion streaks from the GP motion could dominate over the signals provided by conflicting dipole orientation. These results are consistent with the notion that two separate but communicating mechanisms contribute to our perception of GPs which contain conflicting orientation and motion information: (i) perceived GP motion is mediated by spatial motion-direction sensors, in which signals from motion sensors are combined with excitatory input from orientation-tuned sensors tuned to orientations parallel to the axis of GP motion, and (ii) perceived GP orientation is mediated by orientation-tuned sensors which mutually inhibit each other. In the next section, we discuss the neural correlates of form-motion integration in GPs.

## The Neural Correlates of Form-Motion Integration in Glass Patterns

The early visual areas, starting from V1, are known to process local shape information (Hubel and Wiesel, 1968; Wilson, 1991), while the neural circuits that process global shape in GPs are different and vary according to the GP global shape. However, it is widely accepted that high occipitotemporal areas are more sensitive to global shapes rather than local form cues (Kourtzi and Kanwisher, 2001; Murray et al., 2002; Altmann et al., 2003; Kourtzi et al., 2003; Ostwald et al., 2008). There is experimental evidence that local orientation and motion cues in dynamic GPs are processed in the early visual cortex through the formation of motion streaks (Geisler, 1999; Nankoo et al., 2012; Pavan et al., 2017a,b). If so, the first stage of form and motion interaction takes place in the early visual areas. Nonetheless, fMRI studies revealed that form and motion interaction is also present at the level of the extrastriate areas (Braddick et al., 2000; Murray et al., 2003; Apthorp et al., 2013). This means that there are neurons tuned to orientation/form information and are also involved in the processing of the apparent/non-directional motion (Albright, 1984; Lennie, 1998; Kourtzi, 2004; Kourtzi et al., 2005, 2008; Krekelberg, 2005a; [Bibr B103][Bibr B8]; Pavan et al., 2017b). This is supported by human brain imaging and physiological evidence on macaque monkeys.

### Neuroimaging Studies

Human brain imaging studies have been fundamental in the understanding of how the brain perceives and processes GPs. For example, Ostwald et al. (2008) found that static concentric GPs were correlated with activation in higher-order visual areas (ventral lateral regions), whereas translational patterns activated mainly low-level visual areas. However, the authors did not exclude the role of the higher-level visual areas in the processing of translational GPs. The findings indicate a continuum in the integration process from selectivity for local orientation and position cues in early visual areas to selectivity for global form in higher occipitotemporal regions. Additionally, higher-level occipitotemporal areas showed higher classification accuracy than low-level areas in discerning differences in global form structure, consistent with global pooling mechanisms of local signals with similar statistics (i.e., orientation and position). Wilson and Wilkinson (1998) also argued that the global form processing in GPs is supported by neurons in higher occipitotemporal areas that integrate local signals from early visual areas.

Contrasting evidence comes from another fMRI study by Mannion et al. (2009). The authors showed that complex shapes produced by clockwise and counter-clockwise static spiral GPs also activate the visual cortical areas V1, V2, and V3. They found that early visual areas have an important role also in the processing of local orientation features in complex shapes. So far, there are conflicting results on the role of the early visual areas in the processing of complex forms and curvature (i.e., radial, circular, and spiral) from GPs. Hence, additional studies are required to clarify the role of early visual cortex in complex textures.

Krekelberg et al. (2005) performed another fMRI study with dynamic circular and radial GPs and RDKs, which showed that both real/directional motion and apparent/non-directional motion activated the extrastriate area hMT +. hMT + does not seem to distinguish between motion generated by motion cues and motion generated by form cues such as in dynamic GPs, a characteristic that has been called *cue invariance* (Krekelberg et al., 2005). This phenomenon occurs not only with dynamic GPs but also with other types of visual stimuli such as the Enigma optical illusion (Zeki et al., 1993) and the rotating snakes illusion (Kitaoka and Ashida, 2003). Furthermore, they showed that not all the brain cells that respond to real/directional motion are also activated for apparent/non-directional motion. In particular, measuring the selectivity index from the activation measures regarding both the real/directional motion and the implied/non-directional motion, the authors inferred that only 45% of neurons overlapped in the processing of real and apparent motion.

Moreover, using magnetoencephalography (MEG), Liu et al. (2017) investigated the recurrent brain connectivity underlying the processing of global form in dynamic GPs. They found that the perceptual integration of form cues in dynamic GPs was associated with consistent responses in visual areas of the dorsal pathway. The authors showed that perceptual integration induced robust and rapid responses along the dorsal visual pathway in a reversed hierarchical manner. In particular, the authors found that the anterior intraparietal sulcus initially responded within 100 ms, followed by backpropagation of activity to the early visual areas. This experiment showed that the visual areas in the dorsal pathway extract the global form that subsequently guides low-level processing for further refinement of the visual percept. This is an example of the interaction between the ventral and dorsal streams in the processing of global form and motion in dynamic GPs.

### Physiological Studies

Physiological studies on macaque monkeys and computational models revealed that V4 is a cortical area that is activated with circular, radial, and hyperbolic gratings rather than parallel patterns (Kobatake and Tanaka, 1994; Gallant et al., 1996; Wilson et al., 1997). The role of V4 in the processing of concentric forms is further confirmed by lesion studies which found that the disruption of this area causes severe visual form processing deficits (Heywood et al., 1992; Merigan, 1996; Aspell et al., 2006; Dumoulin and Hess, 2007). Moreover, Smith et al. (2007), using extracellular recording in macaque monkeys, showed that V1 and V2 do not show a significant activation to circular and radial static GPs, as found by Mannion et al. (2009) using human neuroimaging, but they are involved in the processing of translational and oriented GPs.

Krekelberg et al. (2003) performed another cell recording study with GPs in two male macaque monkeys. The authors recorded the activity of 168 brain cells in the two hemispheres of the macaques. They observed that motion areas of the superior temporal sulcus (STS) are activated in the processing of dynamic translational GPs, suggesting that the STS is sensitive not only to motion cues but also to form cues contained in dynamic GPs. Thus, this area can integrate form and motion signals to create the perception of a coherent visual stimulus.

Albright (1984) recorded the activity of cells in macaques’ MT area and found that this visual area is not only characterized by cells selective to pattern-motion but also by cells sensitive to orientation cues. Indeed, orientation information might be used by hMT + cells as a hint of motion direction (Movshon et al., 1985). Additionally, Lennie (1998) found that while many MT cells in the macaque monkey cortex respond strongly to motion information, they also respond to a smaller degree to orientation.

Overall, the aforementioned studies show that the visual areas of the ventral and dorsal streams continuously interact for the processing of complex dynamic GPs. However, the connection between the dorsal and ventral streams is not only functional but also anatomical. Rockland and Van Hoesen (1999) showed that there are anatomical connections between the ventral and dorsal pathways. A part of the dorsal pathway, the parieto-medial temporal stream runs to the caudal area of the inferior parietal cortex reaching the hippocampus and the parahippocampal area that is a part of the ventral pathway. Moreover, the lateral parts of the ventral and dorsal pathways are connected by the posterior arcuate fasciculus and the vertical occipital fasciculus. Besides the anatomical connection between the two pathways, a broad interest is upon the functional interplay between the two streams that still deserves to be explored (Yeatman et al., 2014; Weiner et al., 2017).

To conclude, GPs revealed to be a useful category of visual stimuli suitable to increase the knowledge regarding the neural substrates of the interaction between the two streams. GPs have been widely used in basic research to understand the visual functions; however, considerable findings come also from clinical-based research with GPs that showed interesting results regarding the functioning of the ventral and dorsal streams and the processing of form and motion.

## Clinical Applications of Glass Patterns

Several clinical studies (McKendrick et al., 2005; Ditchfield et al., 2006; Spencer and O’Brien, 2006; Tsermentseli et al., 2008; Grinter et al., 2009, 2010; Taylor et al., 2009; Brittain et al., 2010; Koldewyn et al., 2010; Rislove et al., 2010; Englund and Palomares, 2012; Palomares and Shannon, 2013; Day and Palomares, 2014; Bakroon and Lakshminarayanan, 2016) used GPs as a tool to assess form and motion processing in individuals with specific disorders such as autism, reading disorders, dyslexia, and schizophrenia. Spencer and O’Brien (2006) investigated whether children with high-functioning autism show a deficit in form and motion integration besides their difficulty in visual processing such as object recognition and global perception associated with a dysfunction of the ventral stream. Autism is a developmental disorder that causes social, visual, and cognitive deficiencies. In their study, the authors compared three groups of participants: (i) developing individuals with high functioning autism; (ii) developing individuals with Asperger syndrome^1^; and (iii) developing individuals without any disorder (control group). In order to measure the detection thresholds of spatial and form coherence in visual stimuli, the authors used static circular GPs with a background composed of random dots. Participants had to indicate on which side (i.e., either left or right) of the screen they saw the coherent pattern. The results showed that children with autism have an impaired perception of form coherence; however, children with Asperger syndrome did not show impairments of low-level form processing and global motion processing. This evidence is in contrast with the classification of the Asperger syndrome among the autistic spectrum disorders (ASD) because it locates the Asperger syndrome at the lighter end of the continuum. Grinter et al. (2009) performed another study on the autism spectrum disorder using static circular GPs and single moving dots to investigate, respectively, global form and motion processing. In particular, the authors compared two groups of participants: (i) individuals who self-reported high levels of autistic traits and (ii) individuals with a diagnosed autism disorder. The results showed that individuals with high levels of autistic traits had difficulties in the processing of global form and motion integration. This confirms the previous finding of Spencer and O’Brien (2006). Bakroon and Lakshminarayanan (2016) argued that individuals with ASD show good performance in static visuospatial tasks and impaired performance in dynamic visual tasks. According to them, individuals with autism spectrum disorders have dorsal stream deficits and atypical visual cortex interconnectivity that causes an alteration of low-level perceptual processing. However, this statement has been challenged by Koldewyn et al. (2010), who found that adolescents with autism do not show dysfunction in the dorsal stream but only deficits in high-level dynamic attentional processes. Therefore, further studies are needed to clarify the relationship between visual perception and attentional processes related to dynamic visual stimuli in individuals with ASD.

Other evidence comes from a study of Palomares and Shannon (2013) on Williams syndrome. Williams syndrome is a rare genetic and neurodevelopmental disease that causes intellectual disability and visuospatial deficits. In this study, the authors aimed to investigate the functioning of the ventral and dorsal streams in individuals with Williams syndrome by using RDKs and static and dynamic circular GPs. They found that Williams syndrome causes a delay in the development of form processing compared to global motion processing, indicating an impairment in the ventral stream but not in the dorsal stream. In contrast, in typical development, global form processing is developed earlier than global motion processing. Therefore, the late development of form processing in individuals with Williams syndrome leads to difficulties in recognizing shapes and objects. Global form and motion perception have also been studied in other disorders such as schizophrenia (Brittain et al., 2010). Brittain et al. (2010) used single moving dots and static radial and circular GPs to test the functioning of the magnocellular and parvocellular pathways in a group of 64 patients with schizophrenia. The performance of the patients revealed an impairment of the early visual processing of form and motion and, consequently, a deficit of both the magnocellular and parvocellular pathways.

Ditchfield et al. (2006) used static circular GPs and dynamic random dots to evaluate the capacity to integrate local cues into global form and motion visual information in migraineurs. Previous TMS studies demonstrated that migraineurs show an altered neural function of the extrastriate visual area V5 compared to non-headache individuals (Battelli et al., 2002; Fierro et al., 2003). This reflected a poor performance in global motion perception (McKendrick et al., 2001; McKendrick and Badcock, 2004). The main aim of Ditchfield et al. (2006) was to assess whether migraineurs have difficulties in processing both global motion and form or just global motion. The results showed, for the first time, that individuals who suffer from migraine have deficits in the perception of global form and motion in the period between the headaches attacks.

The same difficulty in form and motion integration has been found by McKendrick et al. (2005) in individuals who suffered from glaucoma. Glaucoma is a drainage alteration of the fluids of the eyes that causes abnormal pressure levels. This unusual eye pressure damages the optic nerve that is a bridge between the eye and the brain. The interesting result is that in glaucoma problems that arise with complex visual tasks are not predicted by the exams of visual field loss. Specifically, people with glaucoma can have a high level of difficulties in form and motion processing in areas in which the visual field seems to be normal after standard automated perimetry (SAP).

In another study, Rislove et al. (2010) compared the discrimination performance of static translational and circular GPs in adult individuals with strabismic amblyopia. They found that participants had a higher level of difficulty in discriminating static translational GPs than circular GPs. This further suggests that the mechanisms and the cortical visual areas that allow the detection of translational and circular patterns are different. Some authors have also wondered about the relationship between visual form and motion processing in reading fluency (Conlon et al., 2004; Tsermentseli et al., 2008; Englund and Palomares, 2012). Most of the studies focused on the investigation of reading disorders instead of the healthy development of reading skills in typically developing children. Englund and Palomares (2012) filled this gap, assessing the typical development and involvement of the ventral and dorsal streams in regard to fluency in typically developing children of 9 years of age. The authors measured the participants’ performance in two different form and motion tasks using two different types of visual stimuli: rotating RDKs and static circular GPs. They found that accurate reading is correlated with form processing but reading speed is not correlated with motion coherence thresholds. Moreover, a fluent reading was correlated with good processing of visual information such as form and coherent motion, which rely on the ventral and dorsal streams, respectively. Indeed, previous studies on typically developing participants showed that poor reading skills were correlated with poor coherent motion detection (Cornelissen et al., 1998; Conlon et al., 2004). Another study with RDKs and static GPs was conducted by Alnawmasi et al. (2019). They assessed the functioning of the dorsal and ventral streams in mild traumatic brain injury (TBI) patients with single or multiple concussions, who reported vision-related issues. The results contrast with the hypothesis of dorsal stream vulnerability. Individuals with mild TBI report the same level of impairment in the dorsal and ventral streams, consequently the same level of difficulty in the form and motion processing. The authors concluded that an accurate and precise assessment of global form and motion perception is fundamental in the phase of recovery from mild TBI.

In conclusion, clinical-based research revealed to be useful to better understand the neural basis of form and motion processing and the functioning of the ventral and dorsal streams. The aim of future studies is to develop innovative recovery protocols for specific dysfunctions of the ventral and dorsal streams. Recent research points to exploit non-invasive brain stimulation (NIBS) techniques to potentiate specific brain areas and to compensate the dysfunctions of different cortical sites. Indeed, these techniques add knowledge on brain connectivity.

## Form–Motion Interaction Probed With Non-Invasive Brain Stimulation

Contributions in the understanding of the neural basis of form–motion interaction also come from non-invasive brain stimulation (NIBS) studies (Ellison et al., 2007; Laycock et al., 2007; Tang et al., 2015; Cattaneo et al., 2017; Pavan et al., 2017b). NIBS techniques have been widely used in the last 30 years to understand the causal link between human brain activity and behavior by temporarily modulating the neural functions. Brain stimulation techniques aim to establish the causal involvement of a specific brain region in a cognitive or perceptual function and the time course of its involvement (Silvanto, 2013). NIBS techniques increased the brain functions knowledge that correlational techniques, such as fMRI, could not directly assess (Polanía et al., 2018). There are two main groups of NIBS techniques: transcranial magnetic stimulation (TMS) and transcranial electrical stimulation (tES). TMS modulates the neuronal activity through magnetic fields and triggering action potentials, whereas with tES techniques different types of electrical currents can be delivered through the scalp (e.g., direct or alternating current) and modulate the cortical activity.

For example, Pavan et al. (2017b) using repetitive TMS (rTMS), investigated the role of early visual areas (V1/V2) and hMT + in the perception of dynamic and static translational GPs. The authors showed that rTMS applied over early visual areas affected the perception of static GPs, but the stimulation of area V5/MT did not affect the observers’ performance. On the other hand, when rTMS was delivered over either V1/V2 or V5/MT strongly impaired the perception of dynamic GPs. As pointed out by previous neuroimaging studies, early visual areas might be involved in the processing of the spatial structure of GPs, and interfering with the extraction of the global spatial structure also affects the extraction of the motion component, possibly interfering with early form–motion integration and limiting the formation of motion streaks. However, visual area V5/MT is likely to be involved only in the processing of the motion component of dynamic GPs, suggesting that motion and form cues may interact as early as V1/V2. TMS is also an important technique to explore the connections between different cortical areas. For example, Ellison et al. (2007) used a dual-site TMS technique to study the interaction of posterior parietal cortex (PPC) and human motion area hMT + in a visual search task. Although it is known that both brain areas are involved in visual search tasks, less is known about their connections. The results showed that the two brain areas interact in motion/orientation tasks. Indeed, participants’ performance decreased only when TMS was delivered in combination over PPC and hMT +. Further evidence comes from a study by Thompson et al. (2009) who conducted an rTMS study with plaid stimuli to investigate the mechanisms involved in the integration and segregation of motion cues. Plaid stimuli are made by superimposing two moving gratings with different angles. Plaid stimuli can evoke two different precepts: (i) motion in two different directions and (ii) motion in the same direction due to the integration of the two drifting superimposed gratings. In this study, the authors used 1.0 Hz offline rTMS and showed a dissociation between V1 and the extrastriate visual areas (e.g., V3/V3a and V5) in motion integration processing. Indeed, their results indicated that rTMS over V1 enhanced the perception of coherent motion; instead, extrastriate stimulation decreased the coherent motion perception. The double dissociation found between V1 and the extrastriate areas for the perception of plaid motion adds further knowledge about the neural basis of motion perception and how the visual cortex encodes complex visual stimuli such as plaid.

The double dissociation between V1 and hMT + was also investigated by Silvanto et al. (2005). The authors assessed the awareness for moving stimuli by delivering single-pulse TMS over visual areas V1 and hMT +. TMS was delivered in different time windows: after the presentation of the visual stimulus and in correspondence of the visual stimulus. The results showed that the detection of real motion involves the activity of V1 at a later stage compared to hMT +. Finally, they found that projections from hMT + to V1 are fundamental for motion visual awareness. Therefore, this study highlights the existence and the importance of cortico-cortical connections between V1 and hMT + not only for form and motion processing but also for the awareness. This confirms that the ventral and dorsal streams are not segregated as initially proposed by Ungerleider and Mishkin (1982) but are highly interconnected.

Matsuyoshi et al. (2007) showed that hMT + is causally involved in the processing of motion that is generated by form cues. They applied offline rTMS over the left hMT + and the posterior inferior temporal gyrus (IT) to explore the role of these two brain areas in apparent motion processing. rTMS over hMT + significantly affected the perception of apparent/non-directional motion, but rTMS over IT did not have any effect.

Furthermore, in order to investigate the mechanisms underlying form–motion interaction, Tang et al. (2015) used the visual aftereffect which consisted of the adaptation to a static grating that caused a tilted perception of motion direction of the subsequently presented moving stimulus. Additionally, they used transcranial direct current stimulation (tDCS) to manipulate the neuronal activity of visual area V1 to test the role of the primary visual cortex in the processing of orientation information. Participants were adapted for 3 s to a Gabor patch with a spatial frequency of 3 c/deg; after an interstimulus interval of 160 ms, a global dot motion was presented. Participants had to report whether the direction of the pattern was right or left from vertical. The aftereffect was measured considering the repulsion direction from vertical of the global dot motion (the test stimulus) after adaptation to the oriented Gabor patch. Anodal tDCS was used to enhance the activity of V1, positioning the active electrode over the mastoid bone and the reference electrode over Cz. The results showed that anodal tDCS caused a reduction of the visual aftereffect, suggesting that the primary visual cortex has a fundamental role in the processing of orientation information. Moreover, the authors found that the visual aftereffect is elicited adapting to a wide range of orientations which indicates that various types of orientation information influence the perception of motion direction at a later stage (i.e., hMT +). These results were confirmed by a computational model on motion direction in which motion selective neurons were tuned to orientation-sensitive neurons of V1. To conclude, the authors demonstrated that form information influences motion direction processing.

In summary, NIBS techniques are useful to investigate the mechanisms underlying form–motion integration for three reasons: (i) assess the functional specialization of various visual areas, (ii) assess the deployment over time of a specific visual function, and (iii) understand the heterogeneity of spatial and temporal properties of visual units selective to form/orientation and motion (Silvanto, 2013).

## Discussion

The percept of apparent/non-directional motion induced by dynamic GPs has been used as a tool to investigate the neural basis of form and motion interaction (Lennie, 1998; Or et al., 2010; Mather et al., 2013; Pavan et al., 2017a,b). The existence of systematic interactions between form and motion is in contrast to the old and long-standing view that considers the dorsal and ventral visual pathways as anatomically and functionally independent (Mishkin et al., 1983).

Ungerleider and Mishkin (1982) proposed the classical view that the ventral and dorsal streams are two distinct and independent pathways that do not communicate with each other and independently process form and motion information to guide actions. The perspective that considers the dichotomy between the ventral and dorsal streams is based on the idea that the brain is modular, therefore characterized by separate and independent modules that correspond to precise brain areas (Burr, 2000). The experimental evidence reported in this review supports the notion that the brain is an interconnected network where cortical areas continuously exchange information (Sporns et al., 2004; Schlösser et al., 2006; Craggs et al., 2007; Rogers et al., 2007). There is psychophysical, physiological, brain imaging, and computational evidence (Braddick et al., 2000; Berzhanskaya et al., 2007; Farivar, 2009; Beck and Neumann, 2010; Or et al., 2010; Mather et al., 2012) that the ventral and dorsal streams are not segregated but interact at several levels of visual processing. For instance, as previously mentioned, coherent motion in a dynamic GP has been linked to the induction of motion streaks (Ross et al., 2000; Krekelberg et al., 2005) that, in other terms, indicates that form cues aid direction discrimination of moving objects.

Subsequently, Goodale and Milner (1992) proposed a model based on the distinction between visual perception versus vision for goal-directed action. The authors aimed at suggesting an innovative view about the functional organization of the ventral and the dorsal streams. They argued that the functional differences of the two streams might be better understood by looking at the outputs mediated by the two pathways (Milner and Goodale, 2006, 2008). The authors stated that although both streams are sensitive, to a different extent, to form and motion signals, the two streams process and transmit input signals in different ways: the ventral stream is specialized to convert visual inputs to the representation of objects’ form and spatial relationships, whereas the dorsal stream controls visually guided actions (i.e., reaching and grasping objects) instead of guiding perception. This view that considers the dorsal stream involved in the vision for action has been confirmed by various neuropsychological, neurophysiological, and human neuroimaging studies (Fogassi et al., 1992, 1996; Clower et al., 1996; Kawashima et al., 1996; Rizzolatti et al., 1996; Robertson et al., 1997; Karnath and Perenin, 2005; Culham et al., 2006). The model of Goodale and Milner (1992) might be better understood if we consider that three different main pathways originate from the dorsal stream: the parieto–prefrontal pathway that is involved in spatial working memory, the parieto–premotor pathway important for visually guided action, and the parieto–medial temporal pathway that is related to spatial navigation. These three pathways that process different types of visuospatial functions might be the precursors of the dorsal stream. In this perspective, the dorsal stream might be considered responsible for visuospatial and motion perception and of visually guided actions (Kravitz et al., 2011).

Freud and Ganel (2015) studied the mechanisms underlying goal-directed action. In their experiment, the task consisted of asking participants to grasp 2-D objects with a rectangular shape shown on a computer screen. Their results were compared with those obtained by Ganel and Goodale (2003) on 3-D object grasping. The authors observed that the representations that rely on 2-D and 3-D objects diverge. 3-D objects grasping is based on analytical representations of the shape of objects, whereas 2-D objects grasping is based on holistic representations. In conclusion, 2-D objects are not a reliable proxy for real-object representation, especially for visually guided actions.

In another study, Freud et al. (2017) investigated the idea that objects’ shape could be processed by the dorsal stream (Theys et al., 2015). The authors found that the lateral regions of the ventral stream and the posterior area of the dorsal stream were both activated in relation to shape processing. Furthermore, they showed that the ventral and dorsal streams have a close topographical organization such as similar connectivity to other cortical areas of the brain. Their results challenged the model initially proposed by Ungerleider and Mishkin (1982). In another study of Freud et al. (2018), the authors further explored the role of the dorsal stream in object perception. In this study, they used an interocular suppression technique, the continuous flash suppression (CFS), which makes the visual stimuli undetectable for a few seconds. It is known that the CFS deactivates the ventral pathway while the dorsal pathway activity remains operative (Fang and He, 2005; Almeida et al., 2008; Sakuraba et al., 2012; Han et al., 2016; Tettamanti et al., 2017). Freud et al. (2018) found that 3-D object perception involves the activity of the dorsal stream, showing evidence in contrast to the model of Goodale and Milner (1992): the dorsal stream is not only involved in the processing of the geometric features of the objects that subserve a goal-directed action but also involved in object perception itself. Therefore, the representations of 3-D objects are processed in the dorsal stream (Beer et al., 2009); however, these representations influence perceptual decisions (Fang and He, 2005; Freud et al., 2018; Erlikhman et al., 2019). This suggestion finds support in a study of Konen and Kastner (2008) in which they showed that the ventral and dorsal streams represent similar objects features such as form, size, and viewpoint. However, 3-D processing is not processed similarly in human primates and non-human primates brain (Vanduffel et al., 2002). In fact, Vanduffel et al. (2002) using fMRI found that monkeys did not show a significant activation of intraparietal areas as in humans, while occipital and mid-level extrastriate visual areas revealed similar activation between the two species. Based on the reviewed findings on form–motion integration and object perception, we propose an updated schematic representation from Perry and Fallah (2014), of the main visual areas composing the dorsal and ventral streams, their connections and functions (see Figure 3).

Another type of research that focused on the understanding of form and motion interaction was performed by McCarthy and Grey (2015). They focused on how spatio-temporal form integration (STFI) occurs in the processing of a moving object when only some parts of the object are visible. In particular, they explored the representations of static and rotating objects. In their fMRI study, the authors found that the visual cortical brain areas responsible for STFI are located beyond V1/V2. Furthermore, they showed that despite that motion perception based on form cues activates a wide portion of the visual cortex (Krekelberg et al., 2005), the brain responses related to the updating position of rotating objects were mainly located in visual areas KO and hMT +.

On the other hand, some researchers investigated the interaction of the dorsal and ventral streams not through the study of form and motion processing but through the study of how the brain process objects position. In particular, these studies used the motion-induced position shift (MIPS) (Mather and Pavan, 2009; Anstis and Cavanagh, 2017; Kohler et al., 2017). MIPS is an illusion in which a moving visual stimulus appears shifted in its spatial position. Kohler et al. (2017) found that the encoding of object position takes place in both high-level and early visual areas, indicating that ventral and dorsal streams cooperate to establish the perceived position of an object.

## Conclusion

Several studies reported in this review demonstrate the importance of form and motion integration for the perception of moving objects and complex shapes. The interaction of form and motion processing has deep roots; it starts at the level of the early visual cortex and proceeds toward the motion complex (hMT +). Indeed, the processing of form and motion cues demands recurrent connectivity between low and high visual regions to integrate spatial and temporal characteristics of a moving visual stimulus. In the present review, we also described how form/orientation signals could affect the perception of motion.

Most of the scientific literature that explores form and motion integration, especially in Glass patterns (GPs), is based on correlational methodologies such as fMRI and single-cell recording technique. We suggest deepening the neural basis of dynamic GPs, also using causal-based tools such as TMS and tES that might provide important insights on the recurrent brain connectivity.

## Author Contributions

RD conceptualization and writing-original draft preparation. GC conceptualization, supervision, writing-reviewing and editing. AP supervision, writing-reviewing, editing, figures creation, and curation. All authors contributed to the article and approved the submitted version.

## Conflict of Interest

The authors declare that the research was conducted in the absence of any commercial or financial relationships that could be construed as a potential conflict of interest.

## Footnotes

1 It should be noted that the DSM-V has just a single broad category for autism: autism spectrum disorder (ASD), which replaces all the previous disorders within the spectrum, including Asperger’s disorder.
